# Clinical repercussions and epidemiological considerations of supernumerary canines: A 26 case series

**DOI:** 10.4317/medoral.23035

**Published:** 2019-08-18

**Authors:** Jorge Cortés-Bretón-Brinkmann, Natalia Martínez-Rodríguez, Cristina Barona-Dorado, María Martín-Ares, Javier Sanz-Alonso, María-Jesús Suárez-García, Juan-Carlos Prados-Frutos, José-María Martínez-González

**Affiliations:** 1 DDS,MsC, PhD.Assistant Professor of oral prosthodontics and oral surgery School of dentistry, Complutense University of Madrid; Oral Surgeon, Hospital Virgen de La Paloma , Madrid, Spain; 2 DDS,MsC, PhD. Assistant Professor of oral surgery School of dentistry, Complutense University of Madrid; Oral Surgeon, Hospital Virgen de La Paloma , Madrid, Spain; 3 DDS, MsC, PhD. Associate Professor of oral surgery, School of dentistry, Complutense University of Madrid, assistant director Department of Oral and dental Implant Surgery, Hospital Virgen de La Paloma , Madrid, Spain; 4 DDS, MsC, PhD. Assistant Professor of oral surgery School of dentistry, Complutense University of Madrid; Oral Surgeon, Hospital Virgen de La Paloma , Madrid, Spain; 5 DDS, MsC,PhD. Associate Professor of oral surgery, School of dentistry, Complutense University of Madrid. Oral Surgeon, Hospital Virgen de La Paloma , Madrid, Spain; 6 DDS, MD, PhD. Full Professor of oral prosthodontics, School of dentistry, Complutense University of Madrid; 7 DDS, MD, PhD. Full professor of oral surgery, School of dentistry, King Juan Carlos University of Madrid; 8 DDS, MD, PhD, MDV. Full professor of maxillofacial surgery, School of dentistry, Complutense University of Madrid; and the head , Department of Oral and dental Implant Surgery, Hospital Virgen de La Paloma , Madrid, Spain

## Abstract

**Background:**

To establish the prevalence of supernumerary canines (SNC) in a sector of the population of Madrid (Spain), as well possible complications associated with this unusual developmental variation.

**Material and Methods:**

This observational study was performed between 2005 and 2017, among 21,615 patients seeking dental treatment at the Faculty of Dentistry, Complutense University of Madrid (Spain), and at the Virgen de la Paloma Hospital, Madrid (Spain); 22 patients with 26 SNCs were diagnosed. These 22 patients underwent clinical and radiological exploration, registering patient data.

**Results:**

SNCs presented a prevalence of 0.10% of the study population. The supernumerary teeth (SNT) were located in the upper maxilla more frequently (61.54%) than the mandible (38.46%). 69.23% were found to be impacted, also causing the impaction of the permanent canine in 53.85% of these cases. In 15.38%, follicular expansion > 3mm was observed. SNCs were associated with other SNT in only four patients.

**Conclusions:**

Despite of the fact that the SNCs are usually diagnosed casually in the course of radiological exploration, in the present study over half of them (53.85%) caused impaction of the permanent canine. Early diagnosis allows optimal patient management and treatment planning, with intervention at an appropriate time to prevent complications in development and so reduce later treatment need.

** Key words:**Supernumerary canines, case-series, pathology, repercussions, epidemiological considerations.

## Introduction

Hyperdontia, hypergenesis or the presence of supernumerary teeth (SNT) is defined as the existence of teeth in excess of normal dentition (1). Orhan (2) defines the presence of SNT as total teeth numbering over 20 in temporary dentition and over 32 in permanent dentition. It is important to specify any excess tooth/teeth in specific groups of teeth rather than the total dental formula as SNT may coexist in combination with dental agenesis. Classically, multiple hyperdontia has been associated with a number of genetic syndromes and/or congenital craniofacial anomalies (3). Cleidocranial dysplasia, Gardner’s syndrome, Ehlers-Danlos syndrome (4), Ellis van Creveld syndrome (5), Tricho rhino-phalangeal syndrome, Fabry-Anderson syndrome, Down’s syndrome , or cleft lip and/or cleft palate are some of the entities cited in the literature as being related to multiple hyperdontia. Although SNT are a developmental variation, they are relatively frequent (6), (with a prevalence of 0.15-3.9%), but the incidence of supernumerary canines in absence of syndromes/conditions is exceptional. Moreover, an SNC can produce esthetic and functional disorders (7,8).

The etiology of supernumerary teeth within the population is multifactorial with evidence of chromosomal, polygenic, single gene and major environmental influences in this complex etiology; different factors may exert a major influence in different individuals (9). Variation in outcome in a developmental process, such as the formation of the dentition, enables adaptation to different environments. Tooth number, size and shape are determined during the initiation and morphogenetic stages of dental development. The molecular evidence of repetitive signalling throughout initiation and morphogenesis is reflected clinically in the association of anomalies of number, size and shape.

The present study set out to determine the prevalence of supernumerary canines (SNCs) in a sector of the population of Madrid, as well as the possible complications associated with hypergenetic canines.

## Material and Methods

The Ethics Committee of the Complutense University of Madrid approved this retrospective observational study protocol, which was carried out at the Faculty of Dentistry (Complutense University of Madrid, Spain) and the Virgen de la Paloma Hospital (Madrid, Spain). The study included a total of 21,615 patients seeking dental attention at the two study centers between 2005 and 2017. All had panoramic radiographs and periapical or occlusal radiographs obtained at the radiodiagnostic department at the Complutense University.

Whenever the presence of an SNC was discovered, the patient was referred to the Oral Surgery Service for examination. Twenty-two patients were registered presenting 26 SNC. Each patients’ age and sex was recorded on a clinical data sheet, together with the eruption state of the permanent canine and radiological findings from periapical/occlusal and panoramic radiographs. Image-based diagnosis established whether location was maxillary or mandibular, the morphology of the SNC (we establish the differential diagnosis between the primary canine and the supernumerary canine taking into account the specific features of the temporal teeth: colour, crown morphology and root length), the state of the follicular sac, and any association with other SNT.

When all data had been collected, they were entered on a spreadsheet (Ms-Excel) and exported to a statistical analysis software package (SPSS version 25.0 for Windows).

Statistical analysis consisted of univariate analysis (mean, standard deviation, median, etc.) and bivariate analysis to relate different variables and to analyze data variations, using the chi-squared test; statistical significance was established with a confidence interval (CI) of 95%. (*p*<0,05).

## Results

-Univariate analysis 

The study discovered 26 SNCs in 22 patients, representing a prevalence of 0.10 % (CI 95%: -6,095%, 6,295%) of the total population studied (n=21,615). Twelve cases corresponded to men (54.55%) (CI 95%: 34,3%-73,7%), and 10 to women (45.45%) (CI 95%:26,3%-65,7%), establishing a male to female ratio of 1.2:1. The age when patients were first diagnosed ranged from 7 to 77 years, with a mean diagnosis age of 28.0 years (CI 95%:20,2-35,9 years).

In 63,64% (CI 95%:42,87%-81,07%) of cases diagnosis occurred when patients were aged over 20 years.

Of the whole sample, 61.54% (CI 95%: 42,4%-78,2%) of SNCs were located in the upper maxilla, while 38.46% (CI 95%:21,8%-57,6%) were in the mandible. The presence of two SNCs was observed in four patients, representing 18.18% (CI 95%:6,47%-37,64%) of the case sample. Over two thirds (69.23%) of SNCs were found to be impacted (CI 95%:50,2%-84,2%), while 30.77% (CI 95%:15,8%-49,8%) had erupted ( Table 1). The presence of SNCs caused eruption disorders of the permanent canines in 53.85% of cases (CI 95%:35,1%-71,8%). Radiological examination observed that follicle sacs presented expansion >3 mm in only four cases, representing 15.38% of the sample (CI 95%:5,4%-32,5%).

**Table 1:**
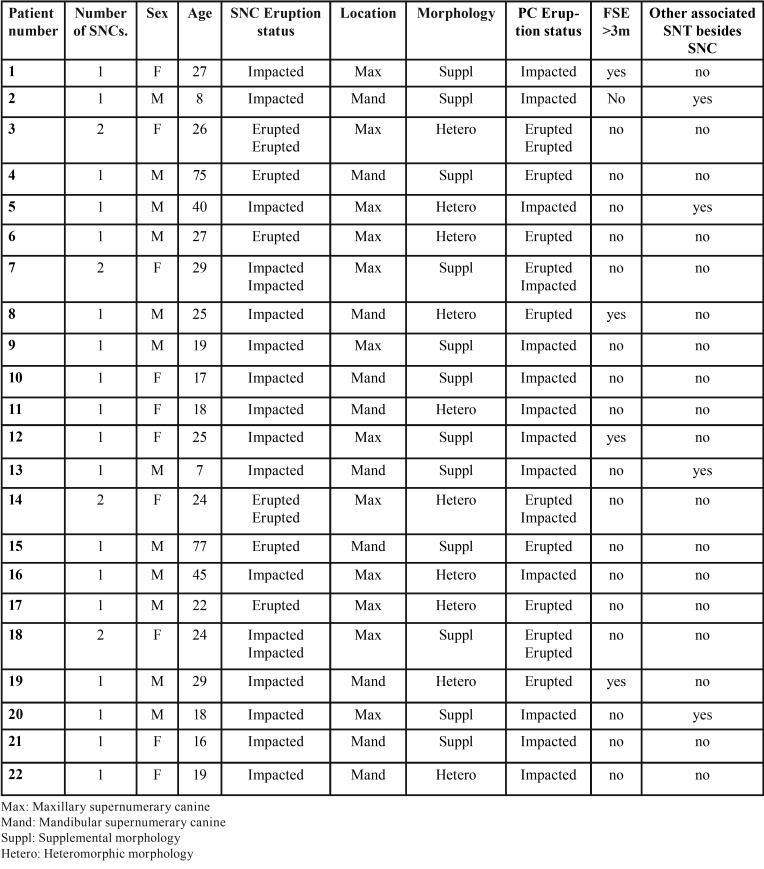
Data of 24 SNCS and their relation with eruption disorders and follicular sac expansion/enlargement >3mm.

Analyzing morphology, 14 SNCs (53.85%) (CI 95%:35,1%-71,8%) presented supplemental morphology, while 12 presented heteromorphic morphology (46.15%) (CI 95%:28,2%-64,9%) representing a ratio of 1/0.86.

Association with other SNT was found in four patients (18.18%) (CI 95%:6,47%-37,64%), noting the presence of two deciduous maxillary lateral incisors and two mandibular incisors.

-Bivariante analysis

Bivariate analysis did not identify any statistically significant differences (*p*<0,05), which may be attributed to the sample size. Nevertheless it was possible to identify and analyze some relations between variables. A relation was identified between patients aged less than 20 years and impaction of the permanent canine (*p*=0.165), causing eruption disorders in every case. Mandibular canines, although few, were more frequently associated with impaction and follicular expansion than maxillary SNCs 80% vs. 50% (*p*=0.350).

## Discussion

The literature includes numerous studies of the prevalence of SNT. Authors such as Nazif *et al.* (10), Davis (11) or Peltola (12) have produced a variety of works of research investigating hypergenesis in general, covering every type of supernumerary tooth (mesiodens, canines, premolars and molars). But studies limited to investigating SNCs alone are scarce and none to date have established a case series (10 or more patients presenting the same phenomenon). As far as the present authors are aware, the present case study of 26 SNCs in 22 patients could be the most extensive published in the literature to date ( Table 1).

The prevalence of SNCs obtained from a population of 21,615 patients, was 0.10% (22 patients. CI 95%: -6,095%, 6,295%). This percentage is slightly higher than the prevalence obtained by other authors (0-0.04%) (11,13,14). The discrepancy can probably be explained by the small number of cases of this type of hyperdontia reported in previous studies. Stafne (15) only found three SNCs among 441 patients with a total of 500 SNT. Stafne considered that denticles and odontomas were found more frequently in the canine region than SNCs.

Rajab and Hamdan (16) diagnosed five SNCs among a total of 202 SNT (2.48%), while Fernández Montenegro *et al.* (17) found four SNCs among 145 SNT (2.75%). But Leco Berrocal *et al.* (18) obtained a higher percentage in their sample (4.17%), although the study only included 24 SNT. Other authors such as Davis (11), Harris and Clark (19) or Burgess (20) not discover any SNCs in their respective studies.

The discovery of hyperdontia is more frequent in men than women in a proportion established by most researchers as around 2:1. This ratio can reach a value of 6.5:1 in the case of Eastern Asian populations (11). The present study did not find significant differences in the male/female proportion, which was 1.2:1. This datum is not comparable to other studies of SNCs, due to a lack of published information.

**Figure 1:**
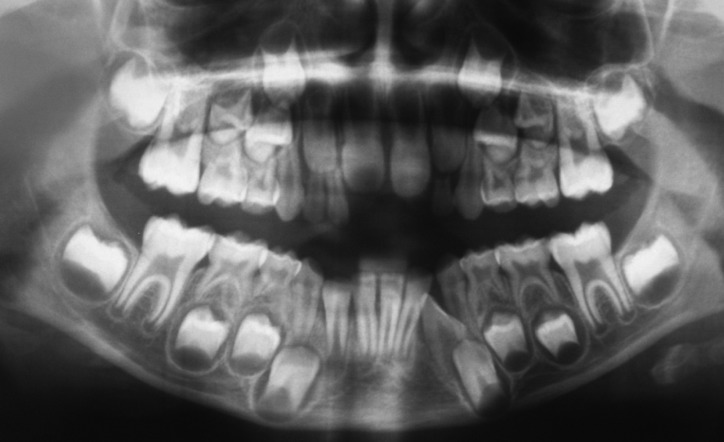
Mandibular supernumerary canine in the third quadrant impeding the eruption of the canine. A supernumerary temporary lateral incisor can also be seen in the first quadrant.

 According to the literature, almost a third of patients with SNT present more than one (1) The presence of multiple SNT (multiple hyperdontia) in absence of associated syndromes and/or systemic conditions is an exceptional finding (21). In the present study, of the 22 patients with SNCs, 14 (63.64%) (CI 95%:42,87%-81,07%) presented a single SNC and four presented two SNCs. Four patients (18.18%) (CI 95%:6,47%-37,64%) presented one SNC with another ST of different dental type (two patients had a supernumerary deciduous maxillary lateral incisor and the other two had supernumerary mandibular lateral incisors) (Fig. 1). Salcido-García *et al.* (6) discovered a case of four SNCs. Türkkahraman *et al.* (7) described a rare case of a twelve-year-old boy with bilateral supernumerary maxillary permanent canines. Hume (22) reported a case of a 17-year-old male with three SNCs (two mandibular and one maxillary).

**Figure 2:**
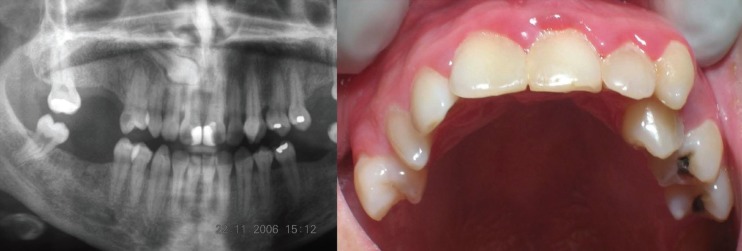
Radiograph and clinical image showing supernumerary canines erupting in the first and second quadrants. In the first quadrant impaction of permanent canine and erupted supernumerary canine. In the second quadrant eruption of both canines (supernumerary and permanent).

According to the literature, SNT occur more frequently in the upper maxilla than the mandible, at a proportion of 5:1 or 10:1 according to different authors (1,23). The present results coincide with most other studies with most of the SNCs located in the upper maxilla (61,54%) (CI 95%:42,4%-78,2%) (Fig. 2). Stafne (15) and Rajab and Hamdan (16) obtained similar results to the present study finding 66.66% and 60.0% (respectively) of canines in the upper maxilla. But Fernández Montenegro *et al.* (17) observed a higher percentage of SNCs in the mandible (75%). Such disparities in results can be explained by the small numbers of SNCs reported in research. As a general rule, the diagnosis of SNT usually occurs as a casual radiological discovery as more often than not these teeth are impacted. Most authors consider that SNT are impacted in between 75 and 80% of cases (1,24). The results of the present study differ slightly as impacted SNCs only represented 69.23% (CI 95%:50,2%-84,2%) of the sample (30.77% were erupted) (CI 95%:15,8%-49,8%). Fernández Montenegro *et al.* (17) found only 50% of SNCs to be impacted, while Salcido-García *et al.* (6) report four impacted SNCs in their study.

SNCs tend to adopt the shape of the original canine. But sometimes they can be of smaller size (8). Of the 26 SNCs in the present study, 14 (53.85%) (CI 95%:35,1%-71,8%) presented supplemental morphology and 12 (46.15%) (CI95%:28,2%-64,9%) heteromorphic morphology. These results more or less coincide with those obtained by Férnández Montenegro *et al.* (17) who observed supplemental morphology in 50% (two out of four SNCs). Others such as Türkkahraman *et al.* (7), Hume (22), Sasaki *et al.* (25), and Stevenson (26) have reported clinical cases of SNCs with supplemental morphology; but Stafne’s study (15) found three SNCs none of which were close to the normal size.

One of the present study’s objectives was to analyze the clinical consequences of SNCs. Türkkahraman *et al.* (7) assert that an ST can produce esthetic and functional disorders. Nadal-Valldaura and Viader Codina (8) maintain that when impacted, SNCs may trigger the symptoms that typify impacted teeth in general. Follicular sac expansion/enlargement >3mm was evaluated as previous studies have considered this a relevant variable. Eliasson *et al.* (27) and Sewerin and von Wovern (28) studied follicular sac expansion >3 mm in impacted mandibular third molars, concluding that although it was not common – 6% and 5.4 % respectively – histological analysis identified the presence of follicular cysts in these cases. In the present study, 15.38% (CI 95%:5,4%-32,5%) of SNCs presented follicular sac expansion >3mm; however, we have no record of later follow-up monitoring to suggest further expansion of the follicular sacs involved. According to the scant literature available, in spite of the hypothetical relation between follicular sac expansion and its evolution into a follicular cyst, this can be considered unusual. Both Shetty and Sandler (29) and Ramakrishna and Lambade (30) report that cystic transformation is infrequent.

**Figure 3:**
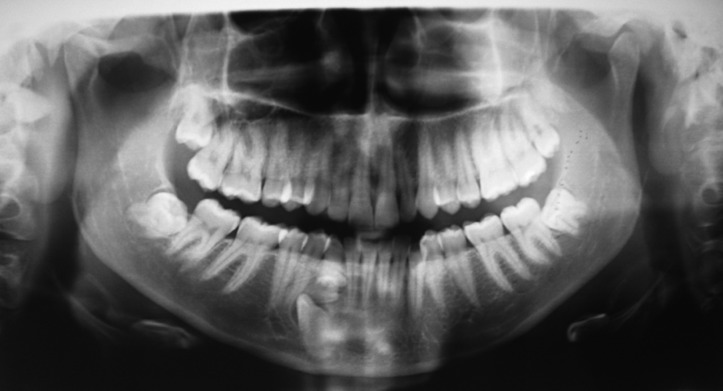
Mandibular supernumerary canine in the fourth quadrant impeding the eruption of the canine.

Over half of the SNCs in the present study (53.85%) (CI 95%:35,1%-71,8%) caused impaction of the permanent canine (Fig. 3). Fernández Montenegro *et al.* (17) identified mechanical-obstructive pathology in two out of four SNCs included in their study, although none presented cystic pathology. It is worth noting that in the present study all SNCs in patients aged younger than 20 years caused eruption disorders (*p*=0.165). However, Sasaki *et al.* (25) reported a case of multiple hyperdontia in the canine and premolar regions in a 14-year-old boy, in which the two SNCs present were not expected to trigger the displacement or mechanical-obstructive pathology of the adjacent teeth.

In any case early diagnosis allows optimal patient management and treatment planning, with intervention at an appropriate time to prevent complications in development and so reduce later treatment need.

It is noteworthy the inherent limitations of our study related with the fact that the 95% CI are large, what can be justified due to the small sample size.

This study analyzed the epidemiological characteristics of a case series of 26 SNCs. SNCs in absence of an associated syndrome or systemic condition are extremely rare and are normally diagnosed casually in the course of radiological exploration. Nevertheless, this type of hyperdontia is associated with a high percentage of pathology.
